# Interhemispheric field-aligned currents at the edges of equatorial plasma depletions

**DOI:** 10.1038/s41598-018-37955-z

**Published:** 2019-02-04

**Authors:** Juan Rodríguez-Zuluaga, Claudia Stolle

**Affiliations:** 1 GFZ, German Research Centre for Geosciences, Potsdam, 14473 Germany; 2 University of Potsdam, Faculty of Science, Potsdam, 14476 Germany

## Abstract

A comprehensive description of electromagnetic processes related to equatorial plasma depletions (EPDs) is essential for understanding their evolution and day-to-day variability. Recently, field-aligned currents (FACs) flowing at both western and eastern edges of EPDs were observed to be interhemispheric rather than anti-parallel about the dip equator, as suggested by previous theoretical studies. In this paper, we investigate the spatial and temporal variability of the FACs orientation using simultaneous measurements of electron density and magnetic field gathered by ESA’s Swarm constellation mission. By using empirical models, we assess the role of the Pedersen conductance in the preference of the FACs to close either in the northern or southern magnetic hemisphere. Here we show that the closure of the FACs agrees with an electrostatic regime determined by a hemispherical asymmetry of the Pedersen conductance. That is, the EPD-related FACs close at lower altitudes in the hemisphere with the highest conductivity. The evidence of this conclusion stands on the general agreement between the longitudinal and seasonal variability of both the conductivity and the FACs orientation.

## Introduction

Interchange instabilities operate in the post-sunset equatorial F region ionosphere due to the mutually perpendicular ambient magnetic field, zonal electric currents, and steep upward plasma density gradients. The evolution of the instabilities generates plasma irregularities in a wide range of scale sizes, from centimeters to hundreds of kilometers. This study discusses observations related to large-scale topside irregularities (few tens to hundreds of kilometers), commonly referred to as equatorial plasma depletions (EPD) or plasma bubbles. EPDs are localized field-aligned regions of depleted plasma that convect after sunset from the bottomside to the topside F region, occasionally reaching altitudes of up to 2000 km or higher. For a thorough review of EPDs and associated irregularities see Hysell^1^.

Extensive work has been done to study the nature of EPDs using airglow imager, ionosonde, global navigation satellite systems, radar, and rocket observations (see Woodman^2^). Nevertheless, the study of electric currents associated with EPDs can only be carried out by using *in situ* magnetic field measurements gathered by low Earth-orbiting satellites, such as the AE-2 and San Marco-D^3^, CRRES^4^, CHAMP^5^, DEMETER^6^, and Swarm^7^. Theoretical and experimental evidence has demonstrated that magnetic perturbations associated with EPDs result from pressure gradient-driven and field-aligned currents mainly (e.g.^8,9^). By means of numerical models, the field-aligned currents (FACs) flowing at the edges of EPDs have been associated with Alfvén waves (e.g.^8,10,11^) and the divergence of zonal currents (e.g.^12–14^). By assuming an ideal ionosphere symmetric about the dip equator, the FACs have been described to flow anti-parallel about the dip equator, i.e., flowing poleward and equatorward at the western and eastern edges of the depletion, respectively (see Fig. 2 of Burke^12^).

**Figure 1:**
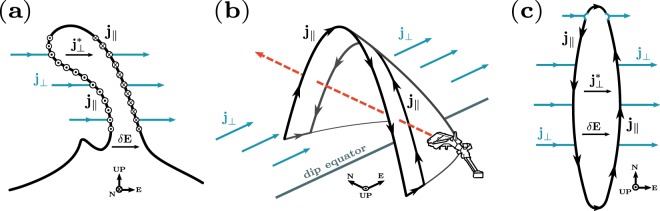
Three views of an EPD wedge-like and its related electric currents. Field-aligned currents $({{\bf{j}}}_{\parallel })$, zonal currents outside (**j**_⊥_) and inside $({{\bf{j}}}_{\perp }^{{\boldsymbol{\ast }}})$ the depletion, polarization electric field (***δ*E**). (**a**) Zonal cross-section at the dip equator. (**b**) Interception with Swarm. (**c**) View from above. The example shows a particular case of ${{\bf{j}}}_{\parallel }$ closing around the southern foot of the depletion. The coordinates depicted correspond to magnetic coordinates.

Recently, Rodríguez-Zuluaga, *et al*.^7^ investigated the direction of the EPD-related Poynting flux using simultaneous measurements of electric and magnetic field gathered by the Swarm satellite mission for a roughly 6-month period in 2014. The authors found that the orientation of the Poynting flux is interhemispheric rather than anti-parallel about the dip equator. It implies that the orientation of the FACs at the edges of EPDs is also interhemispheric. Figure 1 offers a schematic illustration of FACs at the edges of EPDs. It provides three different views of an EPD and its associated electric currents. They refer to a particular case in which the field-aligned currents $({{\bf{j}}}_{\parallel })$ close around the southern foot of the EPD. In detail, Fig. 1a presents a typical zonal cross-section of an EPD at the dip equator as seen from the south. Outside the depletion, zonal currents (**j**_⊥_) are mainly the sum of gravity-driven, Pedersen and inertial currents. Inside the depletion, zonal currents $({{\bf{j}}}_{\perp }^{{\boldsymbol{\ast }}})$ build up to maintain the current continuity through Pedersen and gravity-driven currents. Figure 1b depicts an EPD as a wedge-like structure. This representation can be pictured by mapping the EPD in Fig. 1a to conjugate locations in the northern and southern hemispheres. Figure 1c shows the depletion from above. Following back the work by Rodríguez-Zuluaga, *et al*.^7^, the authors suggest that asymmetry in ionospheric conductivity between both magnetic hemispheres might play a significant role in determining the flow direction of the currents. By using measurements gathered by the C/NOFS satellite mission, Burke, *et al*.^15^ also suggest an interhemispheric current configuration where only one magnetic hemisphere gets disturbed at the bottom side, implying a single Alfvénic disturbance. The idea of an off-equator EPD has been supported using a three-dimensional numerical simulation with electromagnetic features that showed electric fields at the dip equator remotely mapped via Alfvén waves^11^.

In this paper, we make use of an extended dataset of the magnetic field and electron density observations from the Swarm mission to provide a comprehensive analysis of the spatial and temporal variability of the flow configuration of EPD-related FACs. In comparison with climatological predictions of the background ionosphere, we assess the role of the ionospheric conductivity in determining the observed direction of FACs.

## Dataset and Methods

The Swarm constellation mission^16,17^ was successfully launched into a near-polar, circular orbit on 22 November 2013. The mission consists of three identical satellites, Swarm Alpha, Bravo and Charlie, of which two (Alpha and Charlie) currently fly side-by-side at an altitude of about 450 km separated by 1.4° in longitude at the equator. The third satellite (Bravo) orbits at a higher altitude of roughly 510 km. Consecutive orbits of each satellite are separated by approximately 20° in longitude with a local time precession of about 10.8 min/day for Alpha and Charlie and about 10.2 min/day for Bravo, resulting in a complete seasonal-local time coverage after five years. The magnetic and plasma measurements and their related payloads on board Swarm are comprehensively described in Tøffner-Clausen, *et al*.^18^ and Knudsen, *et al*.^19^, respectively. In this study, we use observations of the magnetic field and electron density (N_e_) at a rate of 1 Hz. Thus, N_e_ is decimated from its regular sampling rate of 2 Hz to 1 Hz to match the rate of the magnetic measurements. The study period includes continuous observations of the two parameters from 1 December 2013 to 30 April 2018.

**Figure 2:**
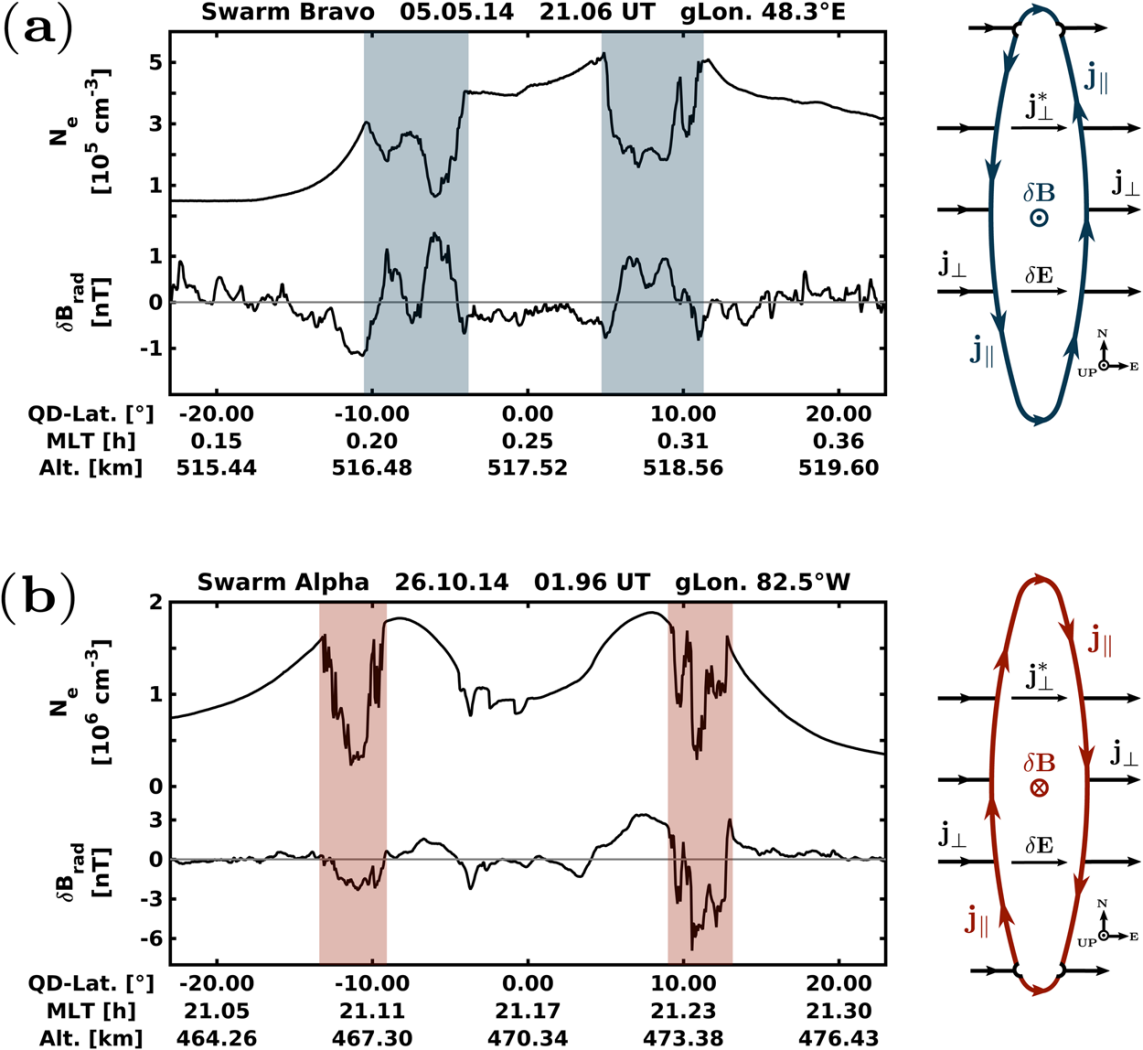
Two passes of Swarm showing two different configurations of interhemispheric FACs. Each panel from top to bottom presents electron density (N_e_) and radial magnetic field component ***δ*B**_**rad**_. To the right, sketch of an EPD see from above describing the corresponding configuration of FACs.

The following statistical analysis is based on a set of EPDs detected by a method similar to the one described in Rodríguez-Zuluaga, *et al*.^7^. Briefly, the background electron density (N_0_) is obtained through a low-pass filter. The procedure is performed over individual passes of Swarm limited to ±30° quasi-dipole latitude and 18–05 magnetic local time (MLT). Then, a preliminary detection results from the residual ***δ***N_e_ = N_e_ − N_0_. At this point, only depletions are identified as EPDs if the percent change between the background and residual (***δ***N_e_/N_0_) is higher than 20%, over a minimum distance of about 23 km (3 s of satellite flight). In this way, we account for decreasing N_0_ along the study period due to the currently declining solar cycle. Finally, for the EPDs to enter into the statistics, they must present distinct magnetic signatures related to FACs. In this respect, we calculate residuals of the magnetic field measurements to a high precision empirical model of the core, crustal and magnetospheric field^20^ and rotate the components into a magnetic-field-aligned coordinate system using the International Geomagnetic Reference Field (IGRF)^21^. In this frame, ***δ*B**_**par**_ points northward along the mean ambient magnetic field, ***δ*B**_**zon**_ is perpendicularly eastward to the magnetic meridian and ***δ*B**_**rad**_ completes the triad pointing vertically outward. Since the EPDs-related FACs have associated transverse magnetic perturbations, their flow direction is deduced solely by ***δ*B**_**rad**_. Thus, for each EPD detected, both ***δ***N_e_ and ***δ*B**_**rad**_ across the depletion must present a correlation coefficient (*cc*) with absolute values larger or equal than 0.6 (|*cc*| ≥ 0.6). This approach guarantees that the considered EPDs have comparable sheets of field-aligned currents flowing in opposite directions at their western and eastern edges. The direction of the FACs is then determined by the sign of *cc* such that for negative values, ***δ*B**_**rad**_ is positive and the FACs flow anti-clockwise along the edges of the depleted structure. If *cc* is positive, ***δ*B**_**rad**_ is negative, and the FACs flow clockwise. Samples for these two cases are provided in Fig. 2 and explained later on.

## Results and Discussion

Figure 2 presents two passes of Swarm, each showing N_e_ and ***δ*B**_**rad**_ as a function of quasi-dipole latitude, magnetic local time and altitude. The two examples reflect typical observations of postsunset EPDs as detected by Swarm^7^. Since EPDs evolve in time to describe a band-shell wedge-like structure^22^, polar-orbiting satellites such as Swarm commonly intersect an EPD in both magnetic hemispheres while observing the background ionosphere in between (see Fig. 1b). In each of the two panels, the EPDs present negative (−0.91 and −0.82) and positive (+0.84 and +0.82) correlation coefficients between ***δ***N_e_ and ***δ*B**_**rad**_, respectively. Since the MLT is not changing considerably along the satellite pass, it is likely that the depletions at each magnetic hemisphere correspond to the same depleted wedge or flux tube. To the right of each panel, a sketch of an EPD seen from above depicts the corresponding configuration of currents. It describes an EPD moving upward with respect to the ambient plasma, as suggested by the depicted eastward polarization electric field (***δ*E**). Outside the depletion the zonal currents (**j**_⊥_) are the sum of gravity-driven, Pedersen and inertial currents mainly. These different current sources vary with altitude, such as the first is more dominant at the peak of the F region, and the other two at the bottom side and topside, respectively. The field-aligned currents $({{\bf{j}}}_{\parallel })$ close anticlockwise around the southern foot (Fig. 2a) or clockwise around the northern foot (Fig. 2b) of the EPD. In each case, at lower altitudes, a small part of the currents must divert around the conjugate foot to maintain the continuity of the current. Finally, both sketches show the perturbed magnetic field ***δ*B**_**rad**_ associated with ${{\bf{j}}}_{\parallel }$.

To distinguished between the two configuration of currents throughout the paper, hereafter, the FACs are referred to as blue and red FACs, corresponding to EPDs with a current system as the one shown in Fig. 2a,b, respectively.

### Spatial characteristics

**Figure 3:**
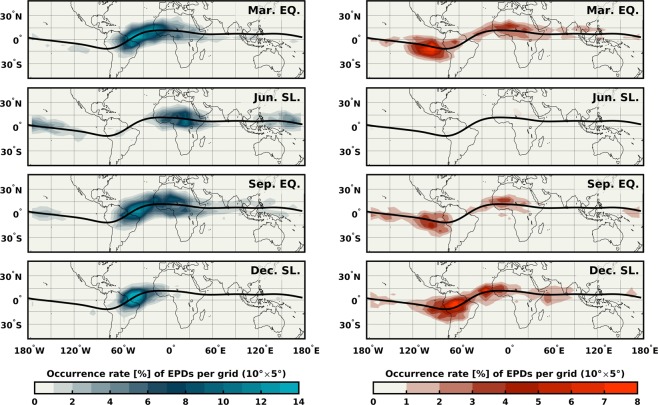
Global and seasonal distribution of EPDs as characterized by their related FACs orientation. Left: EPDs with FACs closing southward (Blue). Right: EPDs with FACs closing northward (Red). Black solid lines indicate the location of the dip equator.

Figure 3 shows the spatial distribution of EPDs as detected by the three Swarm satellites from December 2013 to April 2018. The contour plots represent the occurrence rate within a grid of 10° × 5° in longitude and latitude. The results are further distinguished by the direction of the FACs (left and right panels), and season i.e. March equinox (February, March, and April), June solstice (May, June, and July), September equinox (August, September, and October) and December solstice (November, December, and January). In general, EPDs with FACs closing southward (in blue) occur more frequently than those closing northward (in red).

The longitudinal distribution of EPDs shows a distinct pattern among the different seasons already known from earlier studies. Concerning the FACs direction, the two equinoxes display similar patterns for each current configuration. The blue FACs present a maximum occurrence at about 40°W that decreases toward the African sector. For the red FACs, the occurrence maximizes over the Pacific at about 90°W and over Africa at about 0° longitudes. The red FACs do not overlap with the blue FACs over the American sector but do overlap over the African continent. On the contrary, for the two solstice periods, the FACs present very different patterns. Interestingly, during June solstice all of the EPDs present blue FACs. Differently, December solstice exhibits EPDs with red FACs peaking at about 70°W and 30°W. EPDs with blue FACs instead, maximize around 40°W where the red FACs show a local minimum.

### Temporal characteristics

**Figure 4:**
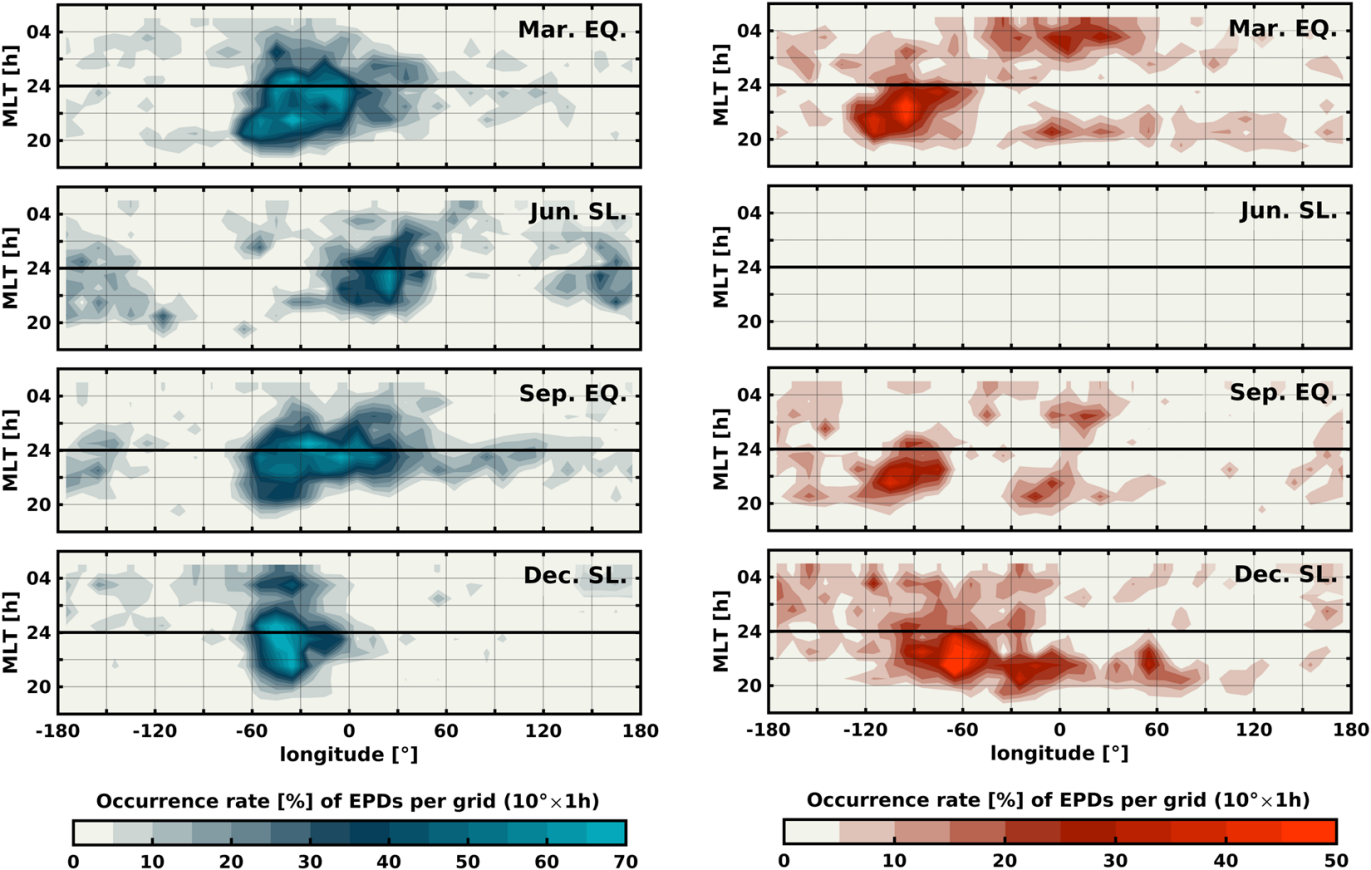
EPDs by season characterized by their related FACs orientation as a function of longitude and magnetic local time. Left: EPDs with FACs closing southward. Right: EPDs with FACs closing northward.

Besides their particular spatial allocation, the EPDs-related FACs also present a longitudinal dependent local time distribution. Figure 4 shows EPDs divided by their related FACs direction as a function of season, magnetic local time (MLT) and longitude. The contours represent the occurrence rate of EPDs within a grid of 10° × 1 h in longitude and MLT. EPDs with blue FACs appear to be persistent throughout the night between 20 and 04 MLT during both equinoxes and December solstice seasons. In June solstice, the EPDs occur before midnight mainly and decay until 02 MLT. The EPDs with red FACs present a preference for pre-midnight hours, however, there is a lower number of post-midnight EPDs over the Atlantic/African sector during equinoctial months. It is interesting to notice that EPDs between about 30°W and 0° present FACs that tend to change direction twice. Before 22 MLT the FACs close northward (red FACs), then they switch to close southward (blue FACs) until 02 MLT when they turn northward again.

### Role of the Pedersen conductance

Previously in this paper, it was mentioned that both the divergence of zonal currents and Alfvén waves are associated with the field-aligned currents at the edges of plasma depletions. Although it implies that the nature of EPDs is a hybrid between electrostatic and electromagnetic modes, it is acceptable to assume that EPDs are predominantly electrostatic in nature (e.g.^8,11^). For the waves, the propagation of the disturbance must be in the direction of the fastest phase velocity i.e., from low to high plasma density. In other words, the waves would propagate towards the hemisphere with the lowest resistivity, reaching an electrostatic state after a few seconds^11^. For the zonal currents, the hemisphere with the highest horizontal conductivity (mainly Pedersen) is where the currents will diverge.

To assess the effect of the conductivity in the preference of the FACs to close around the southern or northern foot of a depletion, we compute the height-integrated Pedersen conductivity (**Σ**_**P**_) as,

1 $${{\rm{\Sigma }}}_{{\rm{P}}}={\int }_{{{\rm{h}}}_{1}}^{{{\rm{h}}}_{2}}{\sigma }_{{\rm{P}}}\,{\rm{dh}},$$

integrating from h_1_ to h_2_ (80 km and 300 km of altitude, respectively). *σ*_P_ is the Pedersen conductivity as given by the conventional formula,

2 $${\sigma }_{{\rm{P}}}=\frac{e{{\rm{N}}}_{{\rm{e}}}}{{\rm{B}}}[\frac{{\omega }_{e}{\upsilon }_{en}}{{\omega }_{e}^{2}+{\upsilon }_{en}^{2}}+\frac{{\omega }_{i}{\upsilon }_{in}}{{\omega }_{i}^{2}+{\upsilon }_{in}^{2}}]$$

where the subscripts *n*, *e*, and *i* represent neutral, electron and ion, respectively. *ω* is the gyrofrequency, *υ* the collision frequency and *e* the electron charge. Here we use the three most abundant ion species along the altitude of integration, i.e., atomic oxygen (O^+^), nitric oxygen (NO^+^) and molecular oxygen $({{\rm{O}}}_{{\rm{2}}}^{+})$. The neutral species considered are also the most abundant in that height range, i.e., O_2_, O, and N_2_. The collision frequencies *υ*_*en*_ and *υ*_*in*_ are calculated as in Schunk and Nagy^23^. The magnetic field is obtained from the IGRF model, the temperatures and densities of the neutrals from the NRLMSISE-00 model^24^ and of the electrons and ions from the IRI model^25^.

**Figure 5:**
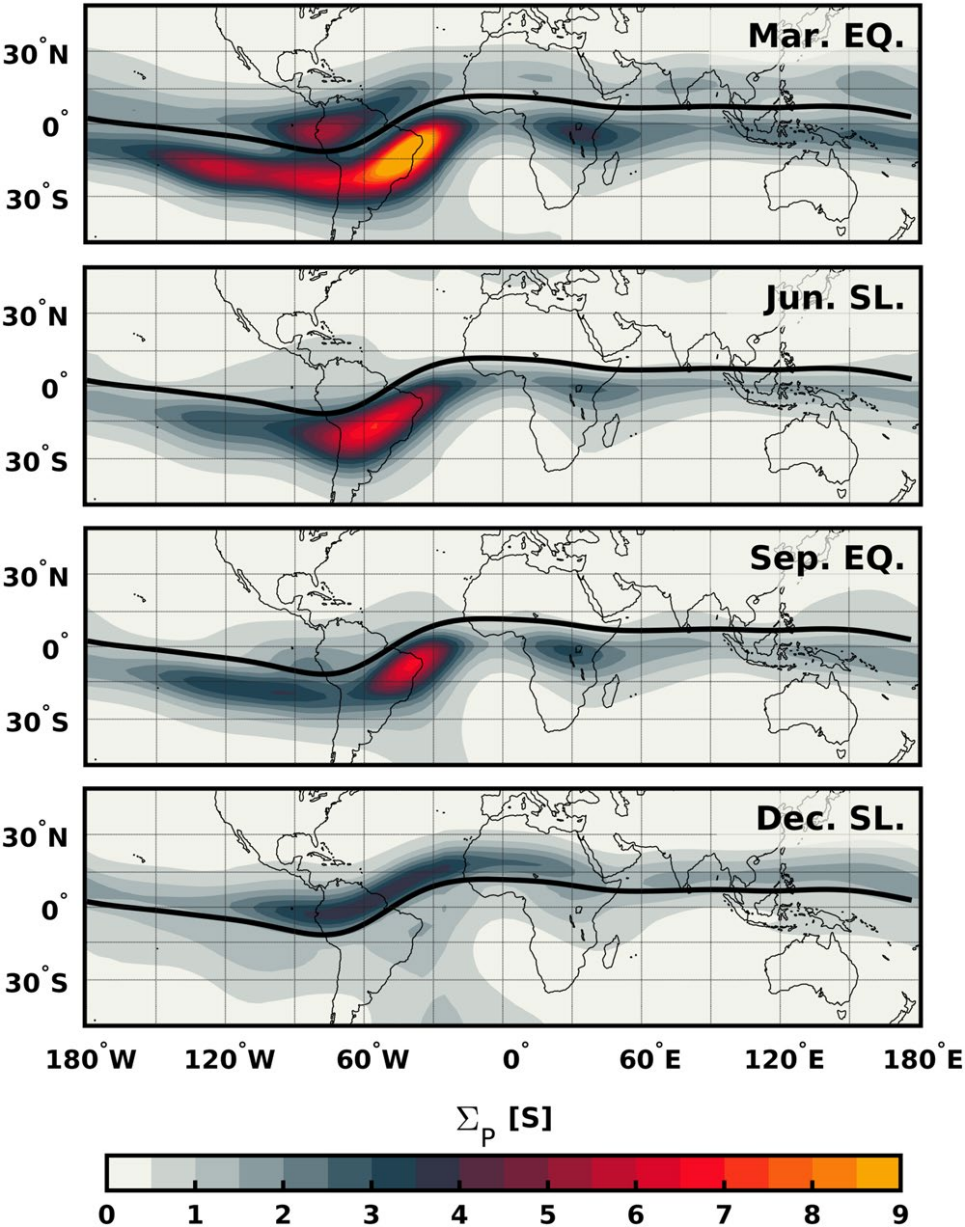
Global maps of the Pedersen conductance derived from IRI and NRLMSISE-00 models for one representative day per season. The integrated altitude ranges from 80 to 300 km. The local time corresponds to 22 hours. Black solid lines indicate the location of the dip equator.

Figure 5 shows global maps of **Σ**_**P**_ for one typical day of each season at 22 **LT** i.e. 21st March, June, September and December of 2015, respectively. In the two equinoctial seasons, the conductance shows similar distributions with larger values at the South Atlantic geomagnetic anomaly (SAA), where the background geomagnetic field intensity drops to about 20.000 nT (compared to about 30.000 nT over the Pacific). At June solstice season, the conductance maximizes in the southern magnetic hemisphere at all longitudes. Conversely, the conductance is more significant in the northern magnetic hemisphere during the December solstice season. Such a distinct feature in the conductance distribution during solstice periods is likely the effect of meridional thermospheric winds blowing from local summer to winter (e.g.^26^). Explicitly, the winds transport the plasma along the field lines pushing the ionosphere upward in the summer hemisphere and downward in the winter hemisphere. Since the relation between the ion-neutral collision frequency and the ion gyrofrequency increases with decreasing altitude, the conductivity turns out to be higher in the winter hemisphere (e.g.^27,28^).

By comparing Fig. 5 with Fig. 3, we find good agreement between the hemispherical asymmetry of **Σ**_**P**_ and the direction to which the FACs close. (1) In both equinoctial seasons, the majority of EPDs detected over the Atlantic (60°W − 0°) present FACs closing in the southern magnetic hemisphere where **Σ**_**P**_ is most significant, e.g., collocated with the SAA. (2) In June solstice, all the identified EPDs exhibit FACs closing southward where **Σ**_**P**_ is the highest at all longitudes. (3) During December solstice, **Σ**_**P**_ is higher in the northern than in the southern magnetic hemisphere, so most of the EPDs present FACs closing northward, except over the SAA where the FACs close southward. Nevertheless, there are other regions where the agreement between the hemispherical distribution of **Σ**_**P**_ and the orientation of the FACs is less evident. In the two equinoctial seasons, the FACs close northward around 90°W though **Σ**_**P**_ does not appear to dominate in the northern magnetic hemisphere at that longitude. We suggest that this might be an effect of zonal thermospheric winds that cannot properly be accounted for through empirical models. During nighttime, eastward winds contribute to the field-aligned transport of plasma in regions with significant magnetic declination. Around the dip equator, the magnetic declination turns positive to the west of 75°W allowing the winds to push down the ionosphere and increase the conductivity in the northern magnetic hemisphere. Another challenging region also in equinox is the one around 0° of longitude. Figure 4 shows that around this longitude the orientation of the FACs switches direction twice, at 22 and 02 MLT. By looking in detail at the variability of **Σ**_**P**_ during those specific local times (not shown), no change is noticed that can explain such behavior, perhaps because of the temporal-scale of the variations which empirical models cannot adequately capture.

## Summary and Conclusions

In this study, we use simultaneous measurements of the magnetic field and electron density from the Swarm mission to assess the spatial and temporal characteristics of the field-aligned currents associated with equatorial plasma depletions. Based on the results reported by Rodríguez-Zuluaga, *et al*.^7^ about the interhemispheric preference of the Poynting flux related to EPDs, two configurations of FACs are possible (see Fig. 2). In general, the FACs are suggested to close around the southern or northern foot of an EPD at the magnetic hemisphere with the highest conductivity. To investigate where, when and which of the two configurations of FACs dominates, we use a continuous dataset starting from December 2013 until April 2018. To evaluate under what conditions one of the two configurations prevails, we compute the Pedersen conductance using empirical models. The main findings and conclusions are summarized as follows.

1. The EPDs-related FACs present a distinct longitudinal pattern among the different seasons. During both equinoxes, the occurrence of EPDs with FACs closing to the southern magnetic hemisphere maximizes about 40°W and decreases toward the African sector. The occurrence of FACs closing northward shows maxima at about 90°W and 0°. Distinctly, the solstice seasons presents contrasting patterns. On June solstice all the EPDs present FACs closing to the southern magnetic hemisphere. During December solstice, the FACs close northward at about 70°W and 30°W mainly and southward at about 40°W where the occurrence of FACs closing northward presents a minimum.
2. The orientation of the FACs exhibits an apparent longitudinal dependent local time distribution. Generally, the EPDs with FACs closing around their southern foot persist throughout the night, while the FACs closing northward present a preference for pre-midnight hours except over the Atlantic/African sector where few of them occur after midnight. An interesting feature is noticed during equinox periods where the EPDs between about 30°W and 0° show FACs switching orientation twice. Before 22 MLT the FACs close northward, then they change southward until 02 MLT when they turn back northward.
3. The Pedersen conductance presents similar spatial distribution in the two equinoxes and opposite in the solstices (see Fig. 5). By comparing with the orientation of the FACs, we found good agreement between the hemispherical asymmetry of **Σ**_**P**_ and the direction to which the FACs close. During both equinoctial periods, the majority of the EPDs detected between about 60°W and 0° exhibit FACs closing southward where **Σ**_**P**_ is more significant (collocated with the SAA). In June solstice, all the EPDs present FACs closing at the southern magnetic hemisphere where **Σ**_**P**_ is the highest at all longitudes. In December solstice, **Σ**_**P**_ maximizes in the northern magnetic hemisphere where most of the FACs close, except over the SAA where the FACs close southward.
4. There are some other regions where the agreement between the distribution of **Σ**_**P**_ and the direction of the FACs is less evident, especially at about 90°W during both equinoctial periods. In such region, the FACs close mostly northward though **Σ**_**P**_ is not more significant than in the southern hemisphere. This observation might be explained by an effect of zonal thermospheric winds that cannot be adequately detected by empirical models.

The interhemispheric FACs reported in this study suggest an electrostatic regime highly determined by a hemispherical asymmetry of the ionospheric conductivity. The FACs are presumed to close at lower ionospheric altitudes through Pedersen currents in the magnetic hemisphere with the highest conductivity. The method of this study does not allow to determine whether the observed FACs originate mainly from the divergence of zonal currents or from Alfvén waves. A detailed investigation is appropriate based on numerical simulations with both electrostatic and electromagnetic characteristics under hemispherical asymmetries of different parameters (e.g., conductivity, plasma density, magnetic field).
